# Supplementary material for: Modeling glioblastoma heterogeneity as a dynamic network of cell states
Source: Mol Syst Biol. 2021 Sep 16;17(9):e10105. doi: 10.15252/msb.202010105 (PMC8444284; doi:10.15252/msb.202010105)
Supplement: Supplementary file 5 — Source Data for Figure 3 [file MSB-17-e10105-s001.zip › Figure3A_sourcedata/GSEA_3065/nosigdb_state2.GseaPreranked.1622718419215/DOROSZKO_U3013MG_CLUSTER_0.html]

Details for gene set DOROSZKO\_U3013MG\_CLUSTER\_0[GSEA]

|  || Dataset | state2 |
| Phenotype | NoPhenotypeAvailable |
| Upregulated in class | na\_neg |
| GeneSet | DOROSZKO\_U3013MG\_CLUSTER\_0 |
| Enrichment Score (ES) | -0.78208524 |
| Normalized Enrichment Score (NES) | -2.3869689 |
| Nominal p-value | 0.0 |
| FDR q-value | 0.0 |
| FWER p-Value | 0.0 |
Table: GSEA Results Summary

  

Fig 1: Enrichment plot: DOROSZKO\_U3013MG\_CLUSTER\_0      
 Profile of the Running ES Score & Positions of GeneSet Members on the Rank Ordered List

  

| PROBE | GENE SYMBOL | GENE\_TITLE | RANK IN GENE LIST | RANK METRIC SCORE | RUNNING ES | CORE ENRICHMENT || 1 | CAMK2N1 |  |  | 408 | 0.198 | -0.0056 | No |
| 2 | CKB |  |  | 647 | 0.157 | -0.0011 | No |
| 3 | SNX1 |  |  | 1873 | 0.070 | -0.1180 | No |
| 4 | MAP2 |  |  | 2271 | 0.055 | -0.1498 | No |
| 5 | SNX22 |  |  | 3545 | 0.023 | -0.2807 | No |
| 6 | RGS2 |  |  | 4038 | 0.015 | -0.3301 | No |
| 7 | NES |  |  | 6455 | -0.025 | -0.5821 | No |
| 8 | MEF2C |  |  | 6861 | -0.033 | -0.6189 | No |
| 9 | NGFR |  |  | 7085 | -0.039 | -0.6352 | No |
| 10 | ANXA1 |  |  | 7265 | -0.044 | -0.6459 | No |
| 11 | F3 |  |  | 7662 | -0.055 | -0.6775 | No |
| 12 | DMD |  |  | 7987 | -0.067 | -0.6992 | No |
| 13 | PGM2L1 |  |  | 8768 | -0.118 | -0.7596 | Yes |
| 14 | BCHE |  |  | 8775 | -0.119 | -0.7377 | Yes |
| 15 | ANXA2 |  |  | 8891 | -0.134 | -0.7244 | Yes |
| 16 | MAP1B |  |  | 8945 | -0.141 | -0.7033 | Yes |
| 17 | CAVIN1 |  |  | 9071 | -0.161 | -0.6860 | Yes |
| 18 | SYT11 |  |  | 9197 | -0.189 | -0.6633 | Yes |
| 19 | GSN |  |  | 9238 | -0.206 | -0.6285 | Yes |
| 20 | IGFBP2 |  |  | 9240 | -0.208 | -0.5891 | Yes |
| 21 | PCSK1N |  |  | 9321 | -0.247 | -0.5506 | Yes |
| 22 | NOVA1 |  |  | 9348 | -0.272 | -0.5017 | Yes |
| 23 | PTPRZ1 |  |  | 9351 | -0.272 | -0.4502 | Yes |
| 24 | VCAN |  |  | 9396 | -0.364 | -0.3857 | Yes |
| 25 | TNC |  |  | 9403 | -0.383 | -0.3135 | Yes |
| 26 | COL6A1 |  |  | 9410 | -0.410 | -0.2362 | Yes |
| 27 | MEG3 |  |  | 9419 | -0.461 | -0.1494 | Yes |
| 28 | IGFBP5 |  |  | 9438 | -0.797 | 0.0002 | Yes |
Table: GSEA details [plain text format]

  

Fig 2: DOROSZKO\_U3013MG\_CLUSTER\_0: Random ES distribution      
 Gene set null distribution of ES for **DOROSZKO\_U3013MG\_CLUSTER\_0**
